# Electroactive Polymeric Composites to Mimic the Electromechanical Properties of Myocardium in Cardiac Tissue Repair

**DOI:** 10.3390/gels7020053

**Published:** 2021-05-01

**Authors:** Kaylee Meyers, Bruce P. Lee, Rupak M. Rajachar

**Affiliations:** Department of Biomedical Engineering, Michigan Technological University, Houghton, MI 49931, USA; kmmeyers@mtu.edu (K.M.); bplee@mtu.edu (B.P.L.)

**Keywords:** composite, cardiac tissue, biomimetic, tissue engineering, conductive

## Abstract

Due to the limited regenerative capabilities of cardiomyocytes, incidents of myocardial infarction can cause permanent damage to native myocardium through the formation of acellular, non-conductive scar tissue during wound repair. The generation of scar tissue in the myocardium compromises the biomechanical and electrical properties of the heart which can lead to further cardiac problems including heart failure. Currently, patients suffering from cardiac failure due to scarring undergo transplantation but limited donor availability and complications (i.e., rejection or infectious pathogens) exclude many individuals from successful transplant. Polymeric tissue engineering scaffolds provide an alternative approach to restore normal myocardium structure and function after damage by acting as a provisional matrix to support cell attachment, infiltration and stem cell delivery. However, issues associated with mechanical property mismatch and the limited electrical conductivity of these constructs when compared to native myocardium reduces their clinical applicability. Therefore, composite polymeric scaffolds with conductive reinforcement components (i.e., metal, carbon, or conductive polymers) provide tunable mechanical and electroactive properties to mimic the structure and function of natural myocardium in force transmission and electrical stimulation. This review summarizes recent advancements in the design, synthesis, and implementation of electroactive polymeric composites to better match the biomechanical and electrical properties of myocardial tissue.

## 1. Introduction

Myocardial infarction (MI) or heart attack occurs when the delivery of oxygen and other nutrients to cardiac muscle cells is hindered, causing myocardial cell death [1]. Since cardiomyocytes have poor regenerative capabilities, damage from heart attack incidents for survivors often leads to the permanent development of acellular, relatively stiff, and non-contractile scar tissue [2]. Loss of myocardial tissue structure and electromechanical function in the damaged region can lead to progressive heart failure and even death. In the United States, there are approximately 805,000 MI incidents a year, 25% of which are repeat MI events for patients [3]. Therefore, many conventional treatment approaches have been developed to either prevent recurrent MI events or restore the mechanical function of the heart while there are little to no options aimed at reverting or repairing damaged myocardium. For example, many pharmaceuticals focus on suppressing future MI events by targeting pathways involved in coronary artery disease, thrombosis of cardiac arteries, or other pathologies that limit the adequate flow of nutrients to heart muscle tissue [1]. Additionally, left ventricular assist devices have been successfully implemented to assist in the mechanical function (i.e., blood pumping) of a failing, damaged heart but this approach has many potential drawbacks such as reducing patient mobility, producing thrombus or embolus in device tubing, and increasing the potential for infection due to the transcutaneous power supply port on the device [4]. A heart transplant procedure is often the last option for a patient with heart failure and limited donor numbers as well as the potential for graft rejection excludes many individuals from receiving a successful transplant [5].

To address the limitations of conventional treatments for scarred heart tissue, alternative approaches for restoring damaged heart function have been developed including stem cell delivery therapies [6]. The principle behind stem cell treatments for cardiac tissue repair includes the injection of patient derived or donor mesenchymal stem cells into the ischemic area of the heart so they can differentiate into cardiomyocytes and become electrically and mechanically coupled to healthy cells in the heart, repairing the damaged region [7]. Limited stem cell survival and the fact that injected stem cells often do not remain in the damaged area of the heart have restricted the effectiveness of this treatment method [8]. Therefore, researchers have designed cardiac scaffolds composed of decellularized extracellular matrix (ECM) and synthetic polymers to aid in the retention and differentiation of stem cells for myocardium repair by acting as a provisional matrix to support cell attachment and infiltration [5,9]. Since these scaffolds are composed of provisional matrix components (i.e., fibrin, collagen, etc.) or synthetic polymers (i.e., polycaprolactone, polyglycercol sebacate, etc.), they often do not match the properties of the native myocardium with respect to modulus (0.02–0.5 MPa) and conductivity (5 × 10^−5^–1.6 × 10^−3^ S/cm), causing limited electromechanical coupling among cells seeded on the scaffolds [10,11]. Failing to emulate the electromechanical conditions of the natural myocardium microenvironment reduces the effectiveness of this approach because myocardial substrate stiffness and electrical stimulation play a vital role in modulating the differentiation, function, and proliferation of myocardiocytes [12,13,14,15]. As a result, scientists and engineers are developing electroactive polymeric composite materials for cardiac scaffolds to mimic native myocardium electromechanical properties [16]. In this review, the recent advancements in the design, synthesis, and implementation of electroactive polymeric composites to better match the biomechanical and electrical properties of myocardial tissue will be summarized. The additional functions of polymeric composites in cardiac tissue repair such as therapeutic delivery, antimicrobial effects, and adhesive element incorporation are also highlighted for relevant examples.

## 2. A Composite Approach to Biomimicry for Cardiac Tissue Engineering

Composites are created by combining two or more materials to achieve a hybrid structure with the chemical and physical qualities of each constituent [17]. More specifically, composites have a matrix phase and one or more reinforcement phases. The matrix phase is generally softer, present in larger volumetric quantities, and contributes to bulk the mechanical properties of the composite [18]. Conversely, reinforcement phases are generally stiffer, present in less volumetric quantities, and can supplement the properties of the matrix phase (i.e., increase material toughness, conductivity, etc.) [18]. There are two major subsets of composite materials: natural and synthetic composites. Common examples of natural composites include wood and bone while materials like carbon fiber reinforced epoxy/nylon and fiberglass are considered synthetic composites.

**Figure 1:**
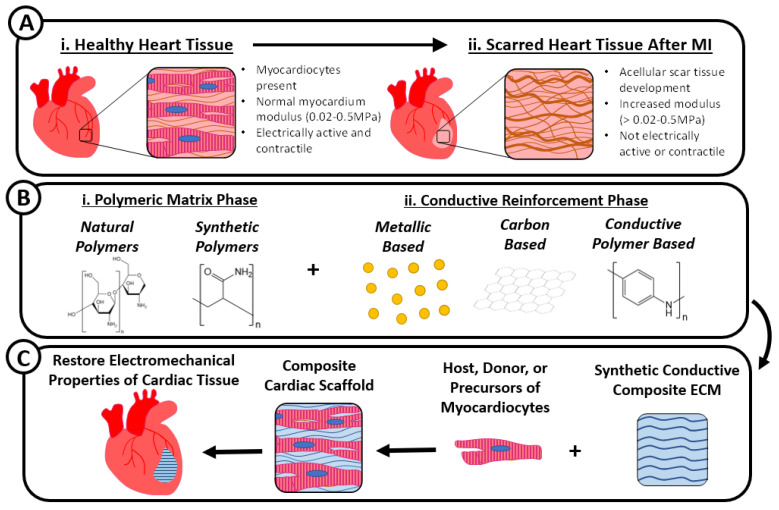
Healthy heart tissue contains myocardiocytes, has a modulus from 0.02 to 0.5MPa, and is electrically active (**A-i**.), whereas heart tissue affected by myocardial infarction (MI) is acellular, scar-like, with an increased modulus, and not electrically active (**A-ii**.). Conductive composites for cardiac tissue engineering are generated by combining natural or synthetic polymers such as chitosan or polyacrylamide (**B-i**.) with conductive elements that are metallic-, carbon-, or polymeric-based such as gold nanoparticles, graphene, or polyaniline (**B-ii**.). These electrically active composites act as a synthetic myocardial extracellular matrix (ECM) by mimicking the electromechanical properties of native myocardium and can be seeded with host, donor, or precursors of myocardiocytes (i.e., mesenchymal stem cells) for cardiac tissue repair (**C**).

Muscle, including myocardial tissue, can also be considered a composite structure since it contains various proteins, fibers, connective tissue, water, nerves, blood vessels, and cells [17]. It is also important to note that muscle tissue presents composite structures at micro and nano hierarchical scales and is anisotropic with respect to its mechanical and electrical properties. Scarred myocardial tissue does not contain all of the constituents present in normal muscle composite tissue and has compromised electromechanical properties (Figure 1A). Therefore, to restore some aspects of the composite structure of damaged muscle tissue while mimicking the proper electromechanical cues, conductive polymer-based composites are being developed. These constructs mimic native cardiac muscle modulus and conductivity by combining a polymeric matrix phase and one or more conductive reinforcement phases (Figure 1B). The polymeric matrix phase can include either natural or synthetic polymers that emulate the stiffness of normal myocardium while the conductive reinforcement phase can contain metallic, carbon, or conductive polymer constituents to give the composite structure a conductivity that matches that of healthy myocardium. During or after the synthesis of composite scaffolds, host or donor myocardiocytes or precursors of myocardiocytes (i.e., mesenchymal stem cells) can be mixed with or seeded onto the composites whereafter cyclic electromechanical stimulation can be applied to emulate the normal myocardial microenvironment and constructs can be implanted or injected into the ischemic areas of damaged myocardial tissue (Figure 1C).

There are many advantages of using conductive composite materials to mimic normal electromechanical properties of the myocardium for cardiac repair and in cardiac in vitro model systems [16]. For example, the conductivity of the composite can be easily tailored by varying the concentration of the conductive reinforcement constituent. Additionally, by altering the crosslinking density of the polymeric matrix phase and/or the concentration of the reinforcement phase, the bulk mechanical properties (i.e., stiffness) of the composite scaffolds can be tuned to better match that of native myocardium. Some reinforcements, such as fibers, can even mimic the anisotropic organization and direction-dependent mechanical and electrical properties of muscle tissue [17]. With a composite approach to biomimicry, it is also possible to include additional constituents that can act as cell and drug delivery vehicles, provide antimicrobial features, or supply adhesive properties which enables conductive polymeric composites that mimic the electromechanical aspects of the myocardium to further augment cardiac tissue repair and regeneration in vivo.

## 3. Reinforcement Methods to Mimic the Electromechanical Properties of Myocardium with Polymeric Composites

### 3.1. Overview of Conductive Reinforcement Types

Composite reinforcement phases can be generated in many forms (i.e., particles, fibers, etc.), sizes (i.e., micro or nano scale), and types (i.e., metallic, carbon, and conductive polymer-based reinforcement) to create electrically active scaffolds for cardiac tissue repair. Each reinforcement technique has distinct advantages and disadvantages that will be described with examples in subsequent sections. An overview of different types of reinforcement techniques for conductive composites in cardiac tissue engineering and their associated conductivity and elastic modulus are listed here (Table 1). The targeted properties of the composites aim to match that of native myocardial modulus (0.02–0.5 MPa) and conductivity (5 × 10^−5^–1.6 × 10^−3^ S/cm) [10,11]. The specific examples of electroactive polymeric composites reviewed were chosen on the basis that they showed successful implementation with in vitro studies.

### 3.2. Metallic-Based Reinforcement

Metallic-based reinforcement in polymeric composites most often occurs in the form of particles, fibers, or sheets. Most metals used in these composite constructs (i.e., gold, silver, and molybdenum) are generally inert, resistant to corrosion, and have very high conductive properties [32]. Additionally, metals can be easily conjugated with antibodies, ligands, or drugs to further promote myocardial tissue regeneration [32]. The amount of the metallic reinforcement constituent in a composite construct has to be tightly controlled though as high concentrations of relatively hard metallics can cause undesired increases in mechanical properties (i.e., stiffness, strength) which can lead to contact damage at the interface of the construct and eccentric host tissue cyclic mechanical loading with heart contractions [33].

**Figure 2:**
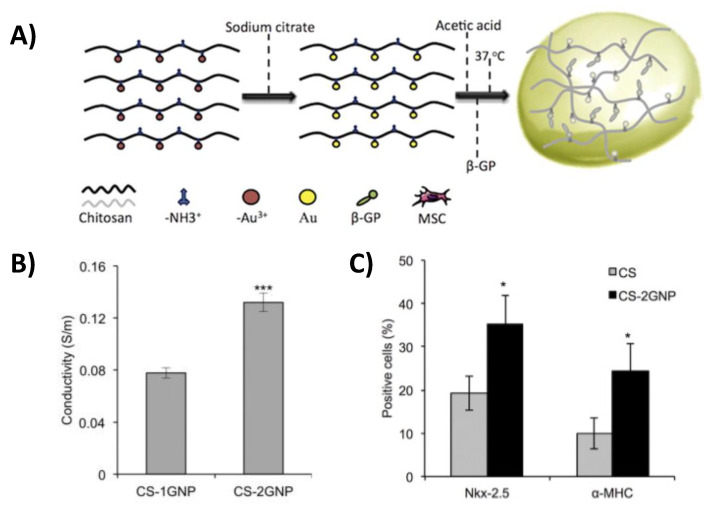
A schematic showing the synthesis of chitosan/gold nanoparticle (CS-GNP) hydrogels (**A**). Dependence of conductivity on gold nanoparticle concentration in chitosan/gold nanoparticle (CS-GNP) composite hydrogels (*** *p* < 0.001) (**B**). Mesenchymal stem cells seeded on constructs containing gold nanoparticles exhibited significantly higher markers of cardiomyocyte differentiation (* *p* < 0.05) (**C**). (CS, CS-1GNP, CS-2GNP, and CS-3GNP indicates 0, 0.5, 1, and 1.5 GNP/CS % w/w, respectively) [19].

One example of using metallic reinforcement in a composite structure for cardiac tissue repair includes chitosan/gold nanoparticle (CS-GNP) hydrogels [19]. These constructs were synthesized by combining chitosan, gold nanoparticles, and β-glycerophosphate (β-GP) while the gold nanoparticles were reduced with a sodium citrate solution (Figure 2A). More specifically, electrostatic interactions between chitosan and β-GP as well as hydrophobic and hydrogen bonding between chitosan chains enabled the gelation of the hydrogel structure. With this approach, Baei et al. was able to show that hydrogels could be generated with a range in stiffness (6.1 × 10^3^–6.8 × 10^3^ Pa) and conductivity (8–13 S/cm) by varying the concentration of gold nanoparticles (Figure 2B). Even with the stiffness of these constructs being slightly less than that of normal myocardium and the conductivity of the composite being much higher than healthy myocardium, markers for the differentiation of mesenchymal stem cells into cardiomyocytes (Nkx-2.5 transcription factor and alpha myosin heavy chain; α-MHC) were significantly higher in samples containing gold nanoparticles (35% Nkx-2.5 and 25% α-MHC positive cells) than pure chitosan hydrogel controls (19% Nkx-2.5 and 9% α-MHC positive cells) (Figure 2C). Another metallic reinforcement example includes thiol-HEMA/HEMA scaffolds that contain gold nanoparticles [21]. These scaffolds were synthesized with photopolymerization and were designed to include micropores that could facilitate cell infiltration. With these constructs, the modulus (0.6 MPa) matched that of normal myocardium very closely and Western blotting showed that rat cardiomyocytes electrically stimulated on the conductive composites had a twofold increase in the expression of connexin-43, a protein that modulates current flow through cardiomyocytes, when compared to cells seeded on non-stimulated scaffolds, indicating that cells were better electrically coupled.

In addition to gold nanoparticles, silver nanoparticles are excellent conductors and inherently antimicrobial, limiting the adhesion and proliferation of harmful bacteria such as *Klebsiella pneumoniae*, *Acinetobacter baumannii*, *Pseudomonas aeruginosa*, and *Escherichia coli* [34]. In one study, silver nanoparticles were suspended in collagen gels to improve the overall conductivity of the material for cardiac repair applications [22]. Neonatal cardiomyocytes electrically stimulated for 24 h on collagen matrices with silver nanoparticles had a two-fold increase of connexin-43 levels when compared to samples that did not contain silver nanoparticles which demonstrates that cells seeded and electrically stimulated on collagen matrices with silver nanoparticles were better electrically coupled. Furthermore, these same gels promoted two times the amount of macrophage infiltration of the M2 phenotype than unpolarized (M0) and M1 phenotype macrophages which indicates that the scaffolds elicited more of a wound healing response rather than an inflammatory one. Another recent metallic reinforced composite for myocardium repair includes nylon/molybdenum disulfide (MoS_2_) nanosheets [20]. These scaffolds exhibited a modulus of 3 × 10^6^ Pa and a conductivity of 20 × 10^−6^ S/cm which are physiologically relevant to myocardial tissue. With real-time PCR, it was found that mouse embryonic cardiac cells seeded on nylon/MoS_2_ sheets expressed significantly higher gene markers of myocardiogenic differentiation (200 times more cardiac muscle troponin and 100 times more alpha myosin heavy chain) when compared to nylon only sheets.

### 3.3. Carbon-Based Reinforcement

Carbon-based reinforcement for conductive polymeric composites can be incorporated in the form of fibers, sheets, or carbon nanotubes (CNT). The advantages of this type of reinforcement include the fact that carbon materials (i.e., CNTs, graphene, graphene oxide, and reduced graphene oxide) can provide adequate thermal, mechanical, and electrical properties as well as a wide range of structural diversity for cardiac regeneration applications [35]. However, CNTs and impurities in graphene materials have shown limited biocompatibility, but the purity, size, concentration, and hydrophilicity of these materials can be adjusted to limit adverse toxicological affects and improve cell viability [36,37]. Graphene-based materials can be used in many medical field applications (i.e., bioimaging, tissue engineering, drug delivery, biosensing, etc.) as their structure can be altered to exhibit geometry in 0, 1, 2, or 3 dimensions [37]. Further modification of graphene via oxidation forms a graphene oxide (GO) material that is amphipathic and more easily hydrated compared to graphene, but the conductivity of graphene is greatly diminished when oxidized [37]. Therefore, researchers have used reducing agents to generate reduced graphene oxide (rGO) which partially restores the electrical properties of the original graphene material [37].

One example of using carbon-based reinforcement in composites for cardiac tissue engineering includes doped carbon nanofibers in chitosan [24]. These porous scaffolds were prepared through the precipitation of carbon fibers in a chitosan matrix. Carbon fiber reinforcement contributed to the relatively high conductive properties for the composite material (4 S/cm) while the chitosan matrix phase influenced the bulk mechanical properties of the material. Additionally, chitosan is inherently anti-oxidative and therefore can aid in reducing the amount of potentially damaging reactive oxygen species near scarred areas of the heart [19]. The overall stiffness of the chitosan scaffold was significantly increased with the addition of carbon nanofibers since chitosan only constructs exhibited a stiffness of 17.8 × 10^3^ Pa whereas carbon fiber reinforced chitosan had a modulus of 28.1 × 10^3^ Pa. Neonatal rat cardiomyocytes seeded on chitosan scaffolds with and without carbon nanofibers for 14 days exhibited significantly higher gene expression (4 fold increase in atrial natriuretic factor, 5.5 fold increase in α-myosin heavy chain, 4.5 fold increase in β-myosin heavy chain and a 2.5 fold increase in gata binding protein) of cardiomyocyte markers involved in cell contraction and the propagation of electrical signals on scaffolds containing carbon nanofibers when compared to chitosan only controls. These results indicate that cells seeded on the carbon nanofiber/chitosan scaffolds were better electrically and mechanically coupled than cells on chitosan only constructs.

**Figure 3:**
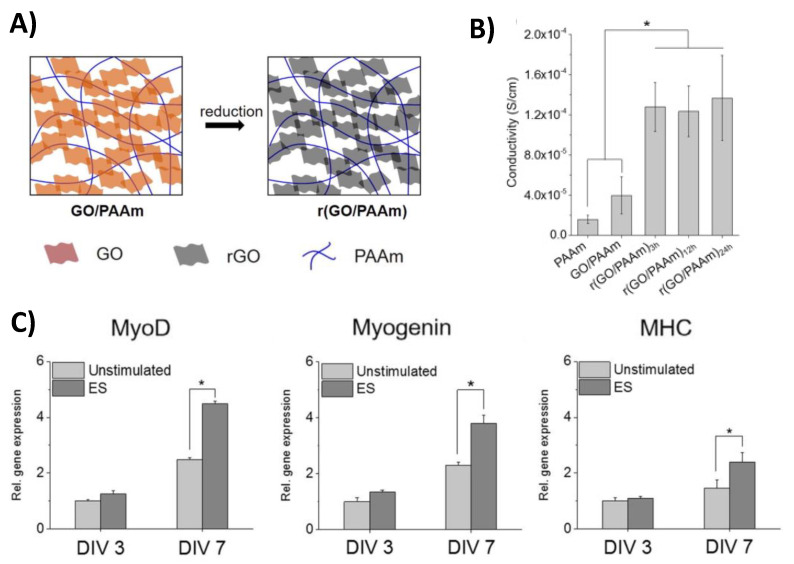
A schematic showing the composition and reduction of graphene oxide/polyacrylamide hydrogels (**A**). Graphene composites reduced for 3, 12, and 24 h (r (GO/PAAm)_3h_, r (GO/PAAm)_12h_, r (GO/PAAm)_24h_) all showed significantly higher conductivity when compared to non-reduced graphene (GO/PAAm) and polyacrylamide only scaffolds (PAAm) (* *p* < 0.05) (**B**). Results from real-time PCR indicate that mouse myoblast cells electrically stimulated (ES) on r(GO/PAAm)_24h_ scaffolds had significantly higher gene expression of cardiomyocyte markers (myoblast determination protein 1: MyoD, myogenin, and alpha myosin heavy chain: MHC) after 7 days of stimulation versus unstimulated scaffolds (* *p* < 0.05) (**C**). No statistical differences in gene expression of cardiomyocyte markers were found in stimulated and unstimulated scaffolds after only 3 days [23].

Carbon-based reinforcement in the form of graphene sheets was also used in polymeric composite materials for muscle tissue repair by creating a reduced graphene oxide/polyacrylamide r (GO/PAAm) hydrogel. This construct was synthesized by crosslinking acrylamide with bisacrylamide in the presence of a graphene oxide solution whereafter the graphene oxide construct was reduced in L-ascorbic acid [23] (Figure 3A). The average stiffness of these hydrogels (50 × 10^3^ Pa) was lower than that of native myocardium, but the conductivity of the reduced graphene oxide/polyacrylamide scaffolds (1.3 × 10^−4^ S/cm) fell within the range of normal myocardium and was significantly higher than non-reduced graphene oxide/polyacrylamide (4.0 × 10^−5^ S/cm) and polyacrylamide (1.6 × 10^−5^ S/cm) constructs (Figure 3B). To evaluate the ability of the hydrogels to promote myocardiocyte differentiation with electrical stimulation, mouse myoblasts were seeded on constructs and stimulated (5 V at 1 Hz for 4 h/day) for 3 or 7 days. Real-time PCR results indicated that the gene expression of myogenic markers (myoblast determination protein 1, myogenin, and alpha myosin heavy chain) were significantly higher for electrically stimulated scaffolds that were reduced for 24 h when compared to unstimulated scaffolds after 7 days (Figure 3C). These results indicated that the conducive aspect of the composite hydrogels could improve myocardiocyte differentiation.

A mussel-inspired carbon-reinforced composite hydrogel composed of chitosan/dopamine/graphene oxide (CS-DA-GO) enabled supplemental functionality to the construct by providing self-healing and adhesivity abilities in addition to mimicking the electromechanical properties of normal myocardium [28]. This hydrogel formulation was able to generate scaffolds with a range of conductivity based on the concentration of GO. For example, constructs with 0 mg/mL of GO had a conductivity of 0.57 × 10^−3^ S/cm whereas constructs with 1 mg/mL of GO had a higher conductivity of 1.22 × 10^−3^ S/cm. In addition, the inclusion of dopamine in the structure amplifies the composite adhesive and self-healing abilities. There are numerous advantages of having an adhesive structure in composite materials for cardiac tissue engineering including better host/scaffold electromechanical integration and improved ease of application in clinical settings. The adhesive character of the composite hydrogels could also be increased by altering the concentration of GO since the researchers noted that adhesive strength increased from 0.15 MPa to 0.95 MPa with GO content until a concentration of 0.5 mg/mL of GO was reached, whereafter adhesive strength started to decrease. Additionally, when hydrogels were transected and tested in compression, stress–strain curve results indicated that self-healed hydrogels showed a 91% recovery rate when compared to undamaged scaffolds. When evaluating the in vitro spontaneous beating rate of myocardiocytes seeded on chitosan/dopamine/graphene oxide hydrogels, it was found that cells were able to beat at a rate (54 times per minute on constructs that contained 0.5 mg/mL GO) similar to a normal human resting heart (60 beats per minute).

### 3.4. Conductive Polymer-Based Reinforcement 

Some polymers are inherently conductive, such as polyaniline (PANi), polypyrrole (PPy), or poly(3,4-ethylenedioxythiophene) polystyrene sulfonate (PEDOT: PSS) and therefore can be incorporated into polymer blends or copolymers to generate polymeric reinforced composites [38]. Advantages of using conductive polymers for composite material reinforcement include that they exhibit excellent biocompatibility and mimic the mechanical properties (i.e., stiffness) of native myocardium [38]. Additionally, polymeric reinforced composites can be easily loaded with therapeutic agents (i.e., growth factors or angiogenic molecules) to improve outcomes of cardiac tissue repair. However, some conductive polymers have limited solubility and are subject to oxidative degradation under environmental service conditions making it more difficult to maintain electrical conductivity over time [10].

**Figure 4:**
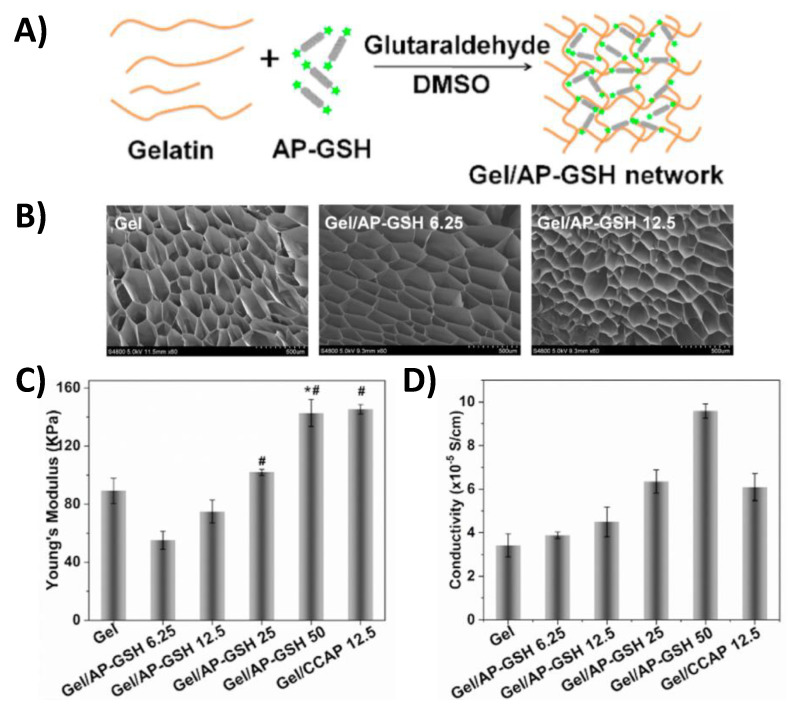
The synthesis of gelatin/aniline pentamer-glutathione composite (Gel/AP-GSH) porous scaffolds (**A**). FESEM images of different w/v% AP-GSH porous scaffolds which allow for better cardiomyocyte infiltration (**B**). The modulus and conductivity of scaffolds containing different concentrations of AP-GSH (**C**,**D**) (* *p* < 0.05 vs. Gel scaffold and ^#^ *p* < 0.05 vs. Gel/AP-GSH 6.25). (Gel = gelatin, Gel/CCAP = gelatin/carboxyl-capped aniline pentamer, and Gel/AP-GSH = gelatin/aniline pentamer-glutathione) [11].

One example of a conductive polymer reinforced composite for cardiac tissue repair includes a gelatin/aniline pentamer-glutathione (Gel/AP-GSH) hydrogel [11]. These constructs were generated by mixing gelatin and aniline pentamer-glutathione which was subsequently crosslinked with a glutaraldehyde solution (Figure 4A). Field Emission Scanning Electron Microscope (FESEM) imaging showed that scaffolds composed of different weight/volume % AP-GSH all contained a porous microstructure which allowed for easier cardiomyocyte infiltration (Figure 4B). By varying the concentration of AP-GSH, a range of physiologically relevant hydrogel stiffness (55.1 × 10^3^–142.7 × 10^3^ Pa) and conductivity (3.4 × 10^−5^–1.0 × 10^−4^ S/cm) could be generated (Figure 4C-D). Since glutathione (GSH) is an antioxidant, this construct also has the potential to reduce local concentrations of reactive oxygen species which could help in improving cardiac tissue repair outcomes. Immunofluorescence results showed the amount of α-actinin, a myocardiocyte differentiation marker, and connexin 43 (Cx-43), an indicator of cardiomyocyte electromechanical coupling, present in rat adipose stem cells seeded on constructs for 7 days was directly dependent on the concentration of AP-GSH in the composite material. Therefore, this result shows that increasing the percentage of the conductive constituent (AP-GSH) in this composite material facilitated the transition of stem cells to a myocardiocyte phenotype.

Poly(glycerol sebacate) (PGS)/collagen/polypyrrole (PPy) hydrogels which incorporate carbonized porous silicon nanoparticles loaded with 3i-1000, a small molecule inhibitor of cardiomyocyte hypertrophy, provide another example of a conductive polymer reinforced composite that could accelerate cardiac tissue repair [31,39]. The PGS component enables the composite to have a stiffness (0.08 × 10^6^ Pa) similar to that of native myocardium while the collagen constituent allowed for cell adhesion. Additionally, the researchers could tune the conductivity of the composite material from 0 to 0.06 S/cm by varying the volume percent of PPy from 0% to 5%. Through the release of 3i-1000 from silicon nanoparticles, these composite hydrogels were also shown to promote cardiomyoblast cell proliferation while inhibiting hypertrophic myocardiocyte responses often associated with MI. Another innovative polymeric-based reinforced composite includes a tetraaniline-polyethylene glycol diacrylate (TA-PEG) and thiolated hyaluronic acid (HA-SH) hydrogel that incorporates plasmid encoding endothelial nitric oxide synthase (eNOS) nanoparticles [30]. The conductivity of these constructs could be altered from 7.08 × 10^−8^ to 2.32 × 10^−4^ S/cm by varying TA-PEG volumetric concentration from 0% to 7.5% which indicates that these hydrogels could match the electrical properties of natural myocardium. In vitro studies were also able to show that the gene encoding eNOS nanoparticles enabled the composite to induce the production of endogenous nitric oxide, a known stimulator of angiogenesis, by a factor of 5 in adipose derived stem cells when compared to untreated controls which is significant because scarred myocardium lacks adequate vasculature and therefore has limited blood supply.

## 4. Future Outlook

Despite promising in vitro study results with electroactive polymeric composites for cardiac tissue repair, there are still some critical barriers to the implementation of these materials in vivo. The largest challenge to implementing these composite materials in vivo is graft-host electromechanical integration [40]. Therefore, the field is transitioning to designing conductive cardiac scaffolds that are injectable, adhesive, and able to cure in situ for better electromechanical interfacing with patient heart tissue without the need for sutures [41,42]. There are many advantages to an adhesive composite material including the fact that sutures create stress concentration and cause damage to otherwise healthy myocardium whereas injectable adhesive materials can adhere and conform to patient-specific geometry in the cardiac tissue microenvironment [41,42]. Some examples of recently developed electrically conductive composite scaffolds that are inherently adhesive include polyaniline-grafted quaternized chitosan and poly(1-pyrenemethyl methacrylate-co-dopamine methacrylamide) [43]. In addition to generating adhesive scaffolds, more in vivo animal studies need to be performed to evaluate the safety and efficacy of composite constructs for repair of scarred myocardium. To further advance the field, improvements in micro and nano technologies have also started to be applied to electroactive polymeric composite materials so that they can mimic the topographical cues present in normal myocardium [44]. Drawing upon developments in other fields related to electrically active tissues (i.e., neuronal tissue) could also potentially help advance the design and development of composite materials used in cardiac tissue engineering.

## 5. Summary

In summary, electroactive polymeric composite materials can be utilized to improve the outcomes of stem cell therapies in cardiac tissue repair. The electromechanical properties of these composites can be tuned to mimic myocardial ECM for cardiac tissue engineering scaffold and in vitro model system applications. To alter the electromechanical properties of composite materials, different types of conductive reinforcement (i.e., metallic, carbon, or conductive polymer) can be utilized. Since composite materials can incorporate many constituents, there is also potential for these constructs to provide additional functionality outside of mimicking electromechanical properties of heart tissue including aiding in the therapeutic delivery of drug and cells, providing antimicrobial features, and being adhesive. Overall, using different types of electroactive polymeric composites offers a unique approach to advancing stem cell therapies in cardiac tissue repair applications, but there are still many tests to be performed and improvements to implement in the field before these materials can potentially be utilized in the clinic.

## Figures and Tables

**Table 1:** Types of reinforcement for conductive cardiac tissue engineering composites (N/A: not available).

Reinforcement Type	Conductive Composite	Conductivity (S/cm)	Modulus (Pa)	Reference
Metallic-Based	Chitosan/gold nanoparticle (CS-GNP) hydrogels	8–13	6.1 × 10^3^– 6.8 × 10^3^	[19]
	Nylon/molybdenum disulfide (MoS_2_) nanosheets	20 × 10^−6^	3 × 10^6^	[20]
	Gold nanoparticles in thiol-HEMA/HEMA scaffolds	1060–1530	0.6 × 10^6^	[21]
	Collagen–silver/gold nanoparticle matrices	0.75 × 10^−4^	N/A	[22]
Carbon-Based	Reduced graphene oxide/polyacrylamide r(GO/PAAm) hydrogel	1.3 × 10^−4^	50 × 10^3^	[23]
	Doped carbon nanofibers in chitosan	4	28.1 × 10^3^	[24]
	Graphene oxide/chitosan (GO/CS) scaffolds	13.4	N/A	[25]
	Polyvinyl alcohol/chitosan/carbon nanofibers (PVA-CS-CNT)	3.4 × 10^−6^	130 × 10^6^	[26]
	Reduced graphene oxide-silver (rGO-Ag) nanocomposites in polyurethane (PU) nanofibers	100 × 10^−6^	210 × 10^6^	[27]
	Chitosan/dopamine/graphene oxide (CS-DA-GO) composite hydrogels	1.22 × 10^−3^	N/A	[28]
Conductive Polymer-Based	Polyaniline (PANI) -poly(glycerol-sebacate) (PGS) composite doped with camphorsulfonic acid	0.018	6 × 10^6^	[10]
	Acid-modified silk fibroin–poly (pyrrole) (AMSF + PPy) substrates	1	7 × 10^6^–200 × 10^6^	[29]
	Gelatin/aniline pentamer-glutathione composite (Gel/AP-GSH)	3.4 × 10^−5^–1 × 10^−4^	55.1 × 10^3^–142.7 × 10^3^	[11]
	Nitric oxide inducing tetraaniline-polyethylene glycol diacrylate (TA-PEG) and thiolated hyaluronic acid (HA-SH) hydrogel	2.32 × 10^−4^	23	[30]
	3i-1000 loaded poly(glycerol sebacate) (PGS)/collagen/ carbonized porous silicon nanoparticle composites	0.06	0.08 × 10^6^	[31]

## Data Availability

Not applicable.

## References

[1] Thygesen K., Alpert J.S., Jaffe A.S., Chaitman B.R., Bax J.J., Morrow D.A., White H.D., Executive Group on behalf of the Joint European Society of Cardiology/American College of Cardiology/American Heart Association/World Heart Federation Task Force for the Universal Definition of Myocardial, I (2018). Fourth Universal Definition of Myocardial Infarction (2018). Circulation.

[2] Kikuchi K., Poss K.D. (2012). Cardiac regenerative capacity and mechanisms. Annu. Rev. Cell Dev. Biol..

[3] CDC (Centers for Disease Control and Prevention) Heart Disease in the United States. https://www.cdc.gov/heartdisease/facts.htm.

[4] Ventricular Assist Device https://www.mayoclinic.org/tests-procedures/ventricular-assist-device/about/pac-20384529.

[5] Baei P., Hosseini M., Baharvand H., Pahlavan S. (2020). Electrically conductive materials for in vitro cardiac microtissue engineering. J. Biomed. Mater. Res. A.

[6] Hashimoto H., Olson E.N., Bassel-Duby R. (2018). Therapeutic approaches for cardiac regeneration and repair. Nat. Rev. Cardiol..

[7] Collins J.M., Russell B. (2009). Stem cell therapy for cardiac repair. J. Cardiovasc. Nurs..

[8] Bolli R., Ghafghazi S. (2017). Stem cells: Cell therapy for cardiac repair: What is needed to move forward?. Nat. Rev. Cardiol..

[9] Zhang J. (2015). Engineered Tissue Patch for Cardiac Cell Therapy. Curr. Treat Options Cardiovasc. Med..

[10] Qazi T.H., Rai R., Dippold D., Roether J.E., Schubert D.W., Rosellini E., Barbani N., Boccaccini A.R. (2014). Development and characterization of novel electrically conductive PANI-PGS composites for cardiac tissue engineering applications. Acta. Biomater..

[11] Li J. (2020). An anti-oxidative and conductive composite scaffold for cardiac tissue engineering. Composites.

[12] Bhana B., Iyer R.K., Chen W.L., Zhao R., Sider K.L., Likhitpanichkul M., Simmons C.A., Radisic M. (2010). Influence of substrate stiffness on the phenotype of heart cells. Biotechnol. Bioeng..

[13] Tallawi M., Rai R., Boccaccini A.R., Aifantis K.E. (2015). Effect of substrate mechanics on cardiomyocyte maturation and growth. Tissue Eng. Part. B. Rev..

[14] Reis L.A., Chiu L.L., Feric N., Fu L., Radisic M. (2016). Biomaterials in myocardial tissue engineering. J. Tissue Eng. Regen. Med..

[15] Saberi A., Jabbari F., Zarrintaj P., Saeb M.R., Mozafari M. (2019). Electrically Conductive Materials: Opportunities and Challenges in Tissue Engineering. Biomolecules.

[16] Palza H., Zapata P.A., Angulo-Pineda C. (2019). Electroactive Smart Polymers for Biomedical Applications. Materials.

[17] Sheffield C., Meyers K., Johnson E., Rajachar R. (2018). Application of Composite Hydrogels to Control Physical Properties in Tissue Engineering and Regenerative Medicine. Gels.

[18] Matthews F.L. (1999). Composite Materials: Engineering and Science.

[19] Baei P., Jalili-Firoozinezhad S., Rajabi-Zeleti S., Tafazzoli-Shadpour M., Baharvand H., Aghdami N. (2016). Electrically conductive gold nanoparticle-chitosan thermosensitive hydrogels for cardiac tissue engineering. Mater. Sci. Eng. C Mater. Biol. Appl..

[20] Nazari H. (2019). Nanofibrous composites reinforced by MoS2 Nanosheets as a conductive scaffold for cardiac tissue engineering. Chem. Select.

[21] You J.O., Rafat M., Ye G.J., Auguste D.T. (2011). Nanoengineering the heart: Conductive scaffolds enhance connexin 43 expression. Nano Lett..

[22] Hosoyama K. (2017). Multi-functional thermo-crosslinkable collagen-metal nanoparticle composites for tissue regeneration: Nanosilver vs. nanogold. RSC Adv..

[23] Jo H., Sim M., Kim S., Yang S., Yoo Y., Park J.H., Yoon T.H., Kim M.G., Lee J.Y. (2017). Electrically conductive graphene/polyacrylamide hydrogels produced by mild chemical reduction for enhanced myoblast growth and differentiation. Acta Biomater..

[24] Martins A.M., Eng G., Caridade S.G., Mano J.F., Reis R.L., Vunjak-Novakovic G. (2014). Electrically conductive chitosan/carbon scaffolds for cardiac tissue engineering. Biomacromolecules.

[25] Jiang L., Chen D., Wang Z., Zhang Z., Xia Y., Xue H., Liu Y. (2019). Preparation of an Electrically Conductive Graphene Oxide/Chitosan Scaffold for Cardiac Tissue Engineering. Appl. Biochem. Biotechnol..

[26] Mombini S., Mohammadnejad J., Bakhshandeh B., Narmani A., Nourmohammadi J., Vahdat S., Zirak S. (2019). Chitosan-PVA-CNT nanofibers as electrically conductive scaffolds for cardiovascular tissue engineering. Int. J. Biol. Macromol..

[27] Nazari H. (2019). Fabrication of graphene-silver/polyurethane nanofibrous scaffolds for cardiac tissue engineering. Polym. Adv. Technol..

[28] Jing X. (2017). Mussel-inspired electroactive chitosan/graphene oxide composite hydrogel with rapid self-healing and recovery behavior for tissue engineering. Carbon.

[29] Tsui J.H., Ostrovsky-Snider N.A., Yama D.M.P., Donohue J.D., Choi J.S., Chavanachat R., Larson J.D., Murphy A.R., Kim D.H. (2018). Conductive Silk-Polypyrrole Composite Scaffolds with Bioinspired Nanotopographic Cues for Cardiac Tissue Engineering. J. Mater. Chem. B.

[30] Wang W., Tan B., Chen J., Bao R., Zhang X., Liang S., Shang Y., Liang W., Cui Y., Fan G. (2018). An injectable conductive hydrogel encapsulating plasmid DNA-eNOs and ADSCs for treating myocardial infarction. Biomaterials.

[31] Zanjanizadeh Ezazi N., Ajdary R., Correia A., Makila E., Salonen J., Kemell M., Hirvonen J., Rojas O.J., Ruskoaho H.J., Santos H.A. (2020). Fabrication and Characterization of Drug-Loaded Conductive Poly(glycerol sebacate)/Nanoparticle-Based Composite Patch for Myocardial Infarction Applications. ACS Appl. Mater. Interfaces.

[32] Mody V.V., Siwale R., Singh A., Mody H.R. (2010). Introduction to metallic nanoparticles. J. Pharm. Bioallied Sci..

[33] Yoo H.Y., Iordachescu M., Huang J., Hennebert E., Kim S., Rho S., Foo M., Flammang P., Zeng H., Hwang D. (2016). Sugary interfaces mitigate contact damage where stiff meets soft. Nat. Commun..

[34] Barras F., Aussel L., Ezraty B. (2018). Silver and Antibiotic, New Facts to an Old Story. Antibiotics.

[35] Maiti D., Tong X., Mou X., Yang K. (2018). Carbon-Based Nanomaterials for Biomedical Applications: A Recent Study. Front Pharmacol..

[36] Smart S. (2006). The biocompatibility of carbon nanotubes. Carbon.

[37] Liao C., Li Y., Tjong S.C. (2018). Graphene Nanomaterials: Synthesis, Biocompatibility, and Cytotoxicity. Int. J. Mol. Sci..

[38] Ning C., Zhou Z., Tan G., Zhu Y., Mao C. (2018). Electroactive polymers for tissue regeneration: Developments and perspectives. Prog. Polym. Sci..

[39] Kinnunen S.M., Tolli M., Valimaki M.J., Gao E., Szabo Z., Rysa J., Ferreira M.P.A., Ohukainen P., Serpi R., Correia A. (2018). Cardiac Actions of a Small Molecule Inhibitor Targeting GATA4-NKX2-5 Interaction. Sci. Rep..

[40] Fidanovski K., Mawad D. (2019). Conjugated Polymers in Bioelectronics: Addressing the Interface Challenge. Adv. Healthc. Mater..

[41] Wu T., Cui C., Huang Y., Liu Y., Fan C., Han X., Yang Y., Xu Z., Liu B., Fan G. (2020). Coadministration of an Adhesive Conductive Hydrogel Patch and an Injectable Hydrogel to Treat Myocardial Infarction. ACS Appl. Mater. Interfaces.

[42] Pourjavadi A., Doroudian M., Ahadpour A., Azari S. (2019). Injectable chitosan/kappa-carrageenan hydrogel designed with au nanoparticles: A conductive scaffold for tissue engineering demands. Int. J. Biol. Macromol..

[43] Pinnaratip R., Bhuiyan M.S.A., Meyers K., Rajachar R.M., Lee B.P. (2019). Multifunctional Biomedical Adhesives. Adv. Healthc. Mater..

[44] Sun S., Shi H., Moore S., Wang C., Ash-Shakoor A., Mather P.T., Henderson J.H., Ma Z. (2020). Progressive Myofibril Reorganization of Human Cardiomyocytes on a Dynamic Nanotopographic Substrate. ACS Appl. Mater. Interfaces.

